# Editorialets

**Published:** 1909-04

**Authors:** 


### Editorialets.

THE "RED BACK'S" PUZZLE CORNER—

Abner Dean of Angel's made a point of order, when
A chunk of old red sandstone took him in the abdomen.
He smiled a kind o' sickly smile and curled up on the floor,
And the subsequent proceedings interested him no more.
Find Abner. —*Bret Harte.*

---

BRAG IS A GOOD DOG.—Holdfast is a better. They live in Chicago. Find Brag.

---

AFTER THE BATTLE.—"You say you were in the battle? I bet you ran, at the first fire," said the Colonel.

"Yes, sir, I did. I'd 'a *run sooner* if I'd a' know'd it was a-comin'," said the tenderfoot.

Find the tenderfoot.

SEVERAL TYPOGRAPHICAL ERRORS occurred on our editorial pages last month, notably, "aline" for "alien"; "magnetism sulphate" for "magnesium sulphate." There were others.

---

DR. J. R. NICHOLS, of Greenville, has been appointed Superintendent of the Southwestern Insane Asylum, vice W. S. Barker.

---

DR. W. F. WAUGH, of Chicago, is collecting material for a paper upon atropine as a hemostatic, and would be obliged to any of our readers who would send him notes of their experience with this remedy. He is particularly anxious to receive adverse reports, as well as those favoring the remedy.

---

WANTED—Slightly used instruments and all kinds of office equipment, in good condition. Fair prices for reliable goods. Distance no object. Write Henderson, 127 East 23d St., New York.

---

FREE SAMPLE of a new patent obstetrical examination cot will be sent to physicians sending card or prescription blank. Other novelties. Address Medical Equipment Company, 127 East 23d St., New York.

---

PROF. G. FRANK LYDSTON, Genito-Urinary Surgeon, Illinois Medical College, will be at Galveston a guest of the Association, and will make "A Chalk Talk on the Prostate." Too late to get paper on the program, so Chase says.

---

*TAKE AN EXTRA* $ or a 2, or a V to Galveston for the "Red Back." You'll get a bill by May 1. Every fellow.

